# Community-based pathogen-specific incidence of influenza-like illness due to respiratory viruses in South-central Vietnam in 2009–2012: after a pandemic of influenza A viruses

**DOI:** 10.1186/s41182-025-00711-x

**Published:** 2025-04-10

**Authors:** Kensuke Takahashi, Shinya Tsuzuki, Minh Nhat Le, Nguyen Hien Anh, Dang Duc Anh, Koya Ariyoshi, Lay-Myint Yoshida

**Affiliations:** 1 Acute & Critical Care Center, Nagasaki University Hospital, Nagasaki, Japan; 2 School of Tropical Medicine and Global Health, Nagasaki University, Nagasaki, Japan; 3 Disease Control and Prevention Center, National Center for Global Health and Medicine, Tokyo, Japan; 4 Faculty of Medicine and Health Sciences, University of Antwerp, Antwerp, Belgium; 5 AMR Clinical Reference Center, National Center for Global Health and Medicine, Tokyo, Japan; 6 Antimicrobial Resistance Research Centre, National Institute of Infectious Disease, Tokyo, Japan; 7 Department of Bacteriology, National Institute of Hygiene and Epidemiology, Hanoi, Vietnam; 8 Department of Clinical Medicine, Institute of Tropical Medicine, Nagasaki University, Nagasaki, Japan; 9 Department of Pediatric Infectious Diseases, Institute of Tropical Medicine, Nagasaki University, Nagasaki, Japan; 10 Graduate School of Biomedical Science, Nagasaki University, Nagasaki, Japan

**Keywords:** ILI, Respiratory virus, Incidence, Healthcare-seeking behaviour, Influenza types A and B

## Abstract

**Background:**

Influenza-like illness (ILI) is one of the most common illnesses caused by various respiratory viruses and directly or indirectly incurs high expenses to households. However, the pathogen-specific incidence and health-seeking behaviour in communities have not been well described.

**Methods:**

A longitudinal cohort study using a self-recorded health calendar among 1000 households was performed in South-central Vietnam from October 2009 to September 2012. Endemic respiratory viruses in the community were monitored using random sampling in public health clinics (polyclinics). The monthly incidence of specific pathogens was calculated using the Bayesian method.

**Findings:**

Among 5,016 household members, 3,687 ILI episodes were reported during the study period. The incidence rate of ILI was 21.7 (95% confidence interval 21.0–22.4) per 1,000 person-months for all ages and highest in children under 2 years with 71.6 (64.7–81.8) followed by 2–4 years with 71.3 (65.8–78.2). Rhinovirus had the highest incidence with 22.5 among the age under 2 years, followed by adenovirus and respiratory syncytial virus (RSV) with 12.5 and 9.9, respectively. Most young children sought treatment from clinics and hospitals, whereas most schoolchildren and adults sought treatment from drugstores. RSV outbreaks significantly increase the number of healthcare visits among children under 2 years, but not in older age groups.

**Interpretation:**

Several surges of ILI were attributed by multiple respiratory viruses. Healthcare seeking patterns were varied among pathogens. Highly transmissible viruses, such as rhinovirus and adenovirus, pose the potential risk of the next pandemic.

**Supplementary Information:**

The online version contains supplementary material available at 10.1186/s41182-025-00711-x.

## Introduction

Influenza-like illness (ILI) is defined by the World Health Organization as a fever accompanied by upper respiratory symptoms, such as cough, sore throat, or rhinorrhoea. It is one of the most common illnesses and causes substantial morbidity and mortality especially in young children and elderly worldwide [1–7].

Most ILI cases are self-limiting and show low mortality rates; however, due to the high incidence among populations, the economic burden of this illness is high. Gerry et al. reported that annual direct costs for hospitalisation and emergency departments due to influenza in the United States were up to $163.25 million and $278.50 million, respectively. [2] Considering not only the direct costs (e.g., fees for healthcare visits and medications) but also the indirect costs (e.g., time off from work or recreation and additional childcare), the total economic burden of ILI is much higher than that estimated only from the direct costs. [2, 7–9]

Various respiratory viruses, including influenza, respiratory syncytial virus (RSV), adenovirus, human metapneumovirus (hMPV), parainfluenza virus, bocavirus, and coronavirus, are known causative pathogens of ILI [3, 7, 11–18]. However, except for specific viruses such as coronavirus disease-2019 (COVID-19), influenza, and RSV, pathogen-specific incidences are not well known due to lack of access to diagnostic tools [7, 18–22]. Several trials for estimating the incidence of respiratory viruses with meta-analysis or modelling, and most etiological surveillance of respiratory viruses have been conducted in hospital settings and focus on severe cases. However, the community burden of ILI due to respiratory viruses has often been neglected and a few community-based surveillances of pathogen-specific incidence of ILI in tropical areas has been conducted [7, 23]. Ngoc et al. reported the household incidence of ILI and identification of 15 respiratory viruses in closed cohort study in Northern Vietnam; however, due to the limitation of study design, age-distributed incidence of ILI nor pathogen-specific incidence could not be reported. [7]

During 2009–2010, a new variant of influenza A (H1N1) virus, namely, the 2009 pandemic H1N1 virus (pH1N1), spread all over the world [24]. Ten years later, we struggled again with the COVID-19 pandemic [25, 26]. The pattern of viruses circulating in the community changes over time [3]. Knowing the characteristics of respiratory viruses is important in preparing for the next pandemic of respiratory viruses.

The aim of this study was to estimate the pathogen-specific incidences of ILI cases in the community in South-central Vietnam after the pH1N1 influenza A pandemic and to reveal the healthcare seeking behaviours of a community during the outbreak of specific viruses.

## Methods

### Health calendar study

We conducted a prospective cohort study in Nha Trang City, Khanh Hoa Province in South-central Vietnam, between October 2009 and September 2012. This is a tropical area with a rainy season, generally during from June to September. Nha Trang consists of 22 communes (administrative divisions similar to municipalities), with 14 in urban areas and 8 in suburban areas. Two communes from urban areas and two from suburban areas were randomly selected, and households were chosen using census data and a random number table, ensuring roughly equal numbers of households from each commune by enrolling only those who provided consent.

We obtained epidemiological information about the households and their members at the beginning of the surveillance period. The sex of the participants was identified as a reported biological factor. Health calendars were distributed to these households to check personal daily events of illness (cough, fever, sore throat, diarrhoea, and body pain) and healthcare-seeking behaviour concerning whether they visited a hospital, public health clinic (polyclinic), private clinic, or community health centre, or just took medication at home. Fever was defined as an axillary temperature of 37.5 °C or higher, as recorded in the household health calendar. Household members were instructed to measure the axillary temperature using a thermometer whenever fever was suspected. Changes in family composition, such as newborn babies, deaths, moving in and out of each household member, and long-staying visitors, were recorded monthly (Supplementary Table 2, Supplementary Fig. 1). Calendars were distributed to each household from October 2009 to September 2012. At the beginning of the study, a trained healthcare worker visited each household and explained how to record the calendar. Healthcare workers visited households every month in the first year and every 2 months in later years to collect calendars and check if the data were properly recorded.

### Polyclinic data

The only two polyclinics serving the four target communes were selected for this study. We collected a list of patients with ILI symptoms who visited polyclinics (nos. 1 and 2) from the four targeted communes between January 2010 and September 2012, including information on age, sex, date of onset, address, and clinical diagnosis. Nasopharyngeal swabs were collected from the first 10 eligible patients < 5 years with ILI symptoms who visited the polyclinics each month. This approach was chosen to monitor circulating viruses in the community in a cost-effective manner. Based on previous studies showing higher viral detection rates in children aged < 5 years, we focused on this age group for virological analysis [7]. Swabs were collected in 1.5 ml microtube containing 600 μL of Skim Milk–Tryptone–Glucose–Glycerol (STGG) media and transported in an icebox to a microbiology laboratory at Khanh Hoa General Hospital (KHGH) on the same day, processed in a standard way, and stored at − 80 °C. RNA was extracted using the QIAamp Viral RNA Mini Kit (#52,906) at the National Institute of Higiene and Epidemiology (NIHE) and respiratory viruses were tested by multiplex polymerase chain reaction (PCR) assays at Nagasaki University [10]. Multiplex PCR was designed to detect 13 respiratory viruses, influenza A and B, RSV, rhinovirus, adenovirus, coronavirus 229E, coronavirus OC43, bocavirus, hMPV, and parainfluenzavirus serotype 1–4 [11, 27]. Outbreak seasons of each virus were defined as 10% or more positive results among tested samples. Panelised logistic multivariate regression models were used to estimate the relationship between outbreak seasons and healthcare facility visits.

### Calculation of pathogen-specific incidence in the community

Pathogen-specific monthly incidences of viruses were calculated as observed ILI cases in the community multiplied by the ratio of circulating viruses obtained in polyclinics. Bayesian methods were used to remove the stochastic effects of sampling:

1 $$ILI_{com}^{k} \,(a,t)_{ } = ILI_{com} \,(a,t) \times p_{poly}^{k} (a,t)$$

where *a* is the age group, *t* is time in months, *k* indicates each respiratory virus, *ILI*_*com*_ is the monthly number of ILI cases in the community and ${ILI}_{com}^{k}$ indicates the pathogen-specific incidence that have not sought medical care (mild ILI cases), and ${p}_{poly}^{k}(a,t)=\frac{{ILI}_{poly}^{k}\left(a,t\right)}{{ILI}_{poly}(a,t)}$ is the proportion of ILI cases infected with pathogen *k* amongst polyclinic patients. Among individuals who visited polyclinics and were diagnosed with ILI each week, we obtained 10 nasopharyngeal swabs from randomly selected patients < 5 years from four target communes. We assumed that the monitored households were representative samples from those four communes, which allowed us to link the circulation of pathogens among ILI patients from the four communes who visited the polyclinics with the number of childhood ILI in those households. To reflect the uncertainty of sampling in ${p}_{poly}^{k}$ in Eq. (1) we employed Bayesian regression techniques and assumed the following:

2 $$ILI_{spoly}^{k} \left( {a,t} \right)\sim Binomial \left( {ILI_{spoly} \left( {a,t} \right), s_{k} \times p_{poly}^{k} \left( {a,t} \right)} \right)$$

where ${ILI}_{spoly}^{k}$ indicates the number of sampled cases of ILI with a positive PCR for pathogen *k* and *s*_*k*_ indicates sensitivity of the test for detecting each pathogen *k*. Equation (2) allows us to derive the posterior distribution of the probability product ${s}_{k} \times {p}_{k}(a,t)$, which, assuming a uniform prior between 0 and 1, is equal to the beta distribution:

$$s_{k} \times p_{poly}^{k} \left( {a,t} \right)\sim Beta\left( {\alpha \left( {a,t} \right),\beta \left( {a,t} \right)} \right)$$

With

$$\alpha \left( {a,t} \right) = ILI_{spoly}^{k} \left( {a,t} \right) + 1$$

3 $$\beta \left( {a,t} \right) = ILI_{spoly} \left( {a,t} \right) - ILI_{spoly}^{k} \left( {a,t} \right) + 1$$

Since we needed ${p}_{k}(a,t)$ in Eq. (1), we had to assume a strong prior on ${s}_{k}$ to avoid identifiability issues on ${p}_{k}\left(a,t\right)$. Using a Bayesian approach, we integrated the uncertainty of the sampling procedure and the test sensitivity (*s*_*k*_) in the regression. Here,* s*_*k*_ represents the uncertainty in the observed test results arising from both the sampling procedure and the inherent test sensitivity of the multiplex PCR assay. By adopting a Bayesian framework, we assumed that the test outcomes (0, 1) followed a beta distribution. This approach allowed us to account for the variability and uncertainty associated with *s*_*k*_, ultimately presenting the results with confidence intervals that reflected this uncertainty. The regression outcome was the posterior distribution of the number of ILI patients infected with pathogen *k* in the community.

The monthly incidence of ILI was described from October 2009 to September 2012, and the pathogen-specific incidence was calculated from January 2010 to September 2012.

### Seasonality of circulating viruses and health seeking behaviour

The outbreak season for each virus, specifically influenza A, influenza B, and RSV, was defined as when the calculated incidence exceeded 10 cases per 1,000 population. For each age group, the odds of visiting any healthcare facilities during the outbreak were calculated. These odds were adjusted based on the outbreak status of other circulating viruses during the same period, allowing for a clearer understanding of health-seeking behavior in relation to specific viral outbreaks.

### Data management

Calendar data were entered and managed using Visual FoxPro^®^ 7. Unknown or unclear information were confirmed via phone calls. Daily data were converted using the following criteria: (a) ILI was defined as a fever and cough or sore throat. We considered that symptoms developed within 7 days belonged to the same episode of illness; (b) if one person visited multiple facilities on the same day, the higher level of facility was applied (order of facility level: KHGH > other hospital > private clinic > polyclinic > Community health centre (CHC) > drug store); and (c) dates when symptoms started and ended were defined as first and final dates of symptom in one chain of symptoms, respectively. Spreadsheet format data were transferred to STATA^®^14 and R^®^ (ver. 4.1.2) for further analysis.

## Results

### Demographic data

The population in the four studied communes was 49,374 according to census data for 2010. Characteristics of selected households and their members at the beginning of the study are shown in Supplementary Table 1. The median number of household members was 4.8 [interquartile range (IQR) 4.0–6.0] persons/house. The median age of participants was 31 (IQR 17–46) years, which became 32 (IQR 18–48) years at the end of surveillance. Half of the sample population graduated from secondary school or higher grades, and at least one person in all households could read and write. The majority of males aged 20–59 years worked as manual workers (49%), followed by white-collar or office workers (23%). The majority of females aged 20–59 years worked as housewives (30%), followed by manual workers (24%) and farmers/fisheries (24%).

At the time of selection, the sample population was 4,696, which increased to 4,716 at the beginning, and 4,755 at the end of the surveillance. Monthly movements of households were monitored (Supplementary Table 2 and Supplementary Fig. 1). Eleven households consisting of 59 members dropped out during the study period; six moved, four refused to participate, and one who lived alone died. There were 148 newborns and 61 deceased members, and 116 members moved in and 93 moved out. Among deceased cases, reasons for deaths were specified in 44 cases: 13 senile cases, 10 malignant cases, 10 brain strokes cases, four accident cases, three heart disease cases, three renal cases, and one asthma case.

### Age-distributed incidence of ILI in the community

**Fig. 1:**
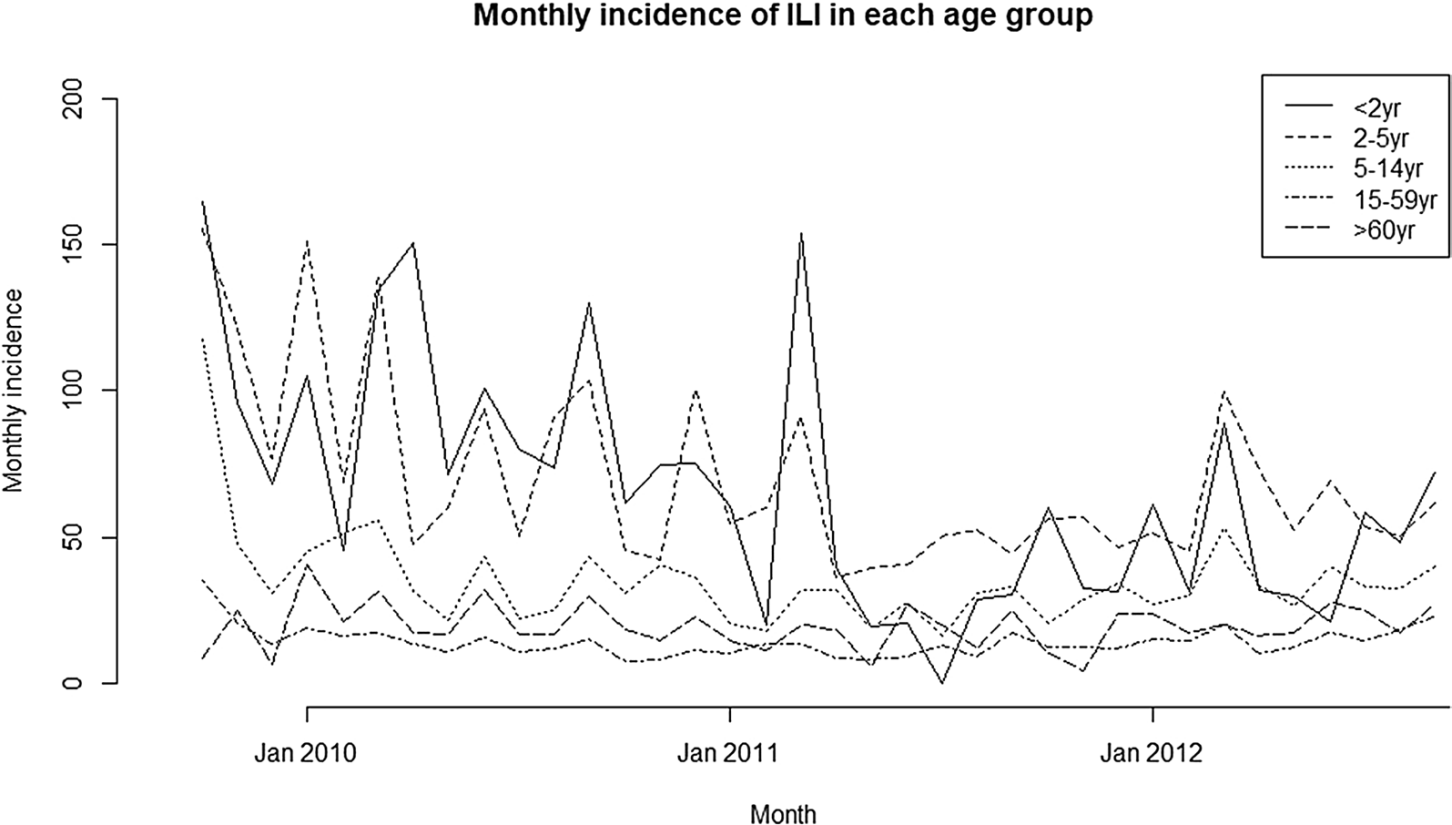
Incidence of ILI per 1000 population-month in each age group. Several surges of ILI symptoms were observed every 2–3 months. Sharp surges of ILI incidence were observed in age group under 2 years (undashed line), which were synchronized with 2–4 years (middle dashed line), 5–14 years (fine dashed line) and > 60 years (rough dashed line). No clear surges were observed in 15–59 years (dotted and dashed lines)

**Fig. 2:**
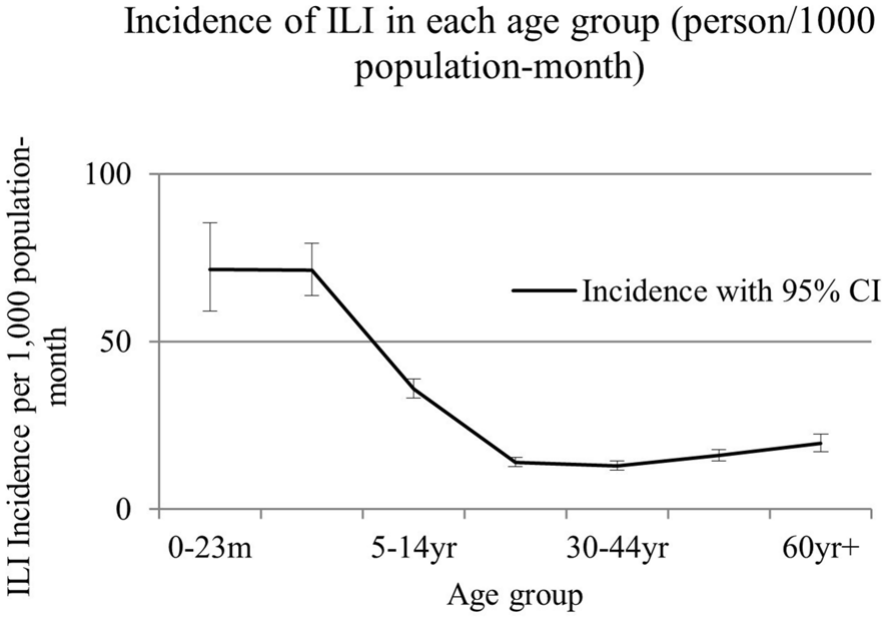
Incidence rate of ILI per 1000 person-months in each age group. Vertical bars indicated 95% confidence intervals (CI). No significant difference was observed between those in age group under 2 years and 2–4 years, 71.6 (95%CI 64.7–81.8) and 71.3(65.8–78.2), respectively. The minimum incidence of ILI was 13.0 (11.9–14.1) in 30–44 years

Several surges in ILI symptoms were observed every 2–3 months (Fig. 1). The incidence rate of ILI was 21.7 (95% confidence interval 21.0–22.4) per 1000 person-months for all ages. The highest incidence was observed for those aged under 2 years with 71.6 [95% confidence interval (95%CI) 64.7–81.8] events/month/1,000 population, followed by those aged 2–4 years with 71.3 (65.8–78.2). The lower incidences were observed in 15–29 years and 30–45 age groups, with 14.0 (13.0–15.2), 13.0 (11.9–14.1) events/month/1,000 population, respectively, and the incidence gradually increased with age (Fig. 2).

### Healthcare-seeking behaviour

Healthcare-seeking behaviour varied significantly by age group. Younger children under 5 years of age most frequently visited public clinics and hospitals, with 22.8% of ILI cases in those under 2 years and 18.2% in those aged 2–4 years seeking care at polyclinics, while private clinics were also commonly used (39.8% and 38.4%, respectively). In contrast, older children and adults increasingly relied on drugstores, with 66.9% of cases in the 5–14 age group and over 79% in adults aged 30 years and above opting for this type of care. Polyclinic visits declined sharply with age, accounting for only 8.1% and 4.2% of cases in the 15–29 and 30–44 age groups, respectively (Table 1).

**Table 1:** Healthcare-seeking behaviour by each age group

Age group (years)	ILI	Healthcare facility, n (%)					
		Drugstores	CHC	Polyclinic	Private clinic	Hospital	Other
< 2	289	114 (39.4)	1 (0.3)	66 (22.8)	115 (39.8)	19 (6.6)	9 (3.1)
2–4	534	253 (47.4)	3 (0.6)	97 (18.2)	205 (38.4)	8 (1.5)	10 (1.9)
5–14	913	611 (66.9)	5 (0.5)	95 (10.4)	174 (19.1)	17 (1.9)	57 (6.2)
15–29	578	463 (80.1)	8 (1.4)	47 (8.1)	62 (10.7)	10 (1.7)	15 (2.6)
30–44	544	433 (79.6)	4 (0.7)	23 (4.2)	70 (12.9)	3 (0.6)	22 (4.0)
45–59	501	413 (82.4)	12 (2.4)	22 (4.4)	49 (9.8)	5 (1.0)	25 (5.0)
60 +	338	270 (79.9)	4 (1.2)	19 (5.6)	26 (7.7)	8 (2.4)	23 (6.8)
Total	3697	2,557 (69.2)	37 (1.0)	369 (10.0)	701 (19.0)	70(1.9)	161 (4.4)

*CHC* community health centre. Drugstores include going to a drugstore or taking drugs stocked in their households. A polyclinic is a public clinic, where the consultation fee is basically free for children under 6 years, whereas private clinics offer paid services. Participants reported as “hospital” when they required admission regardless of the length of stay. “Other” included nowhere to go, traditional medicine or herbal medicine

### Viral results

**Fig. 3:**
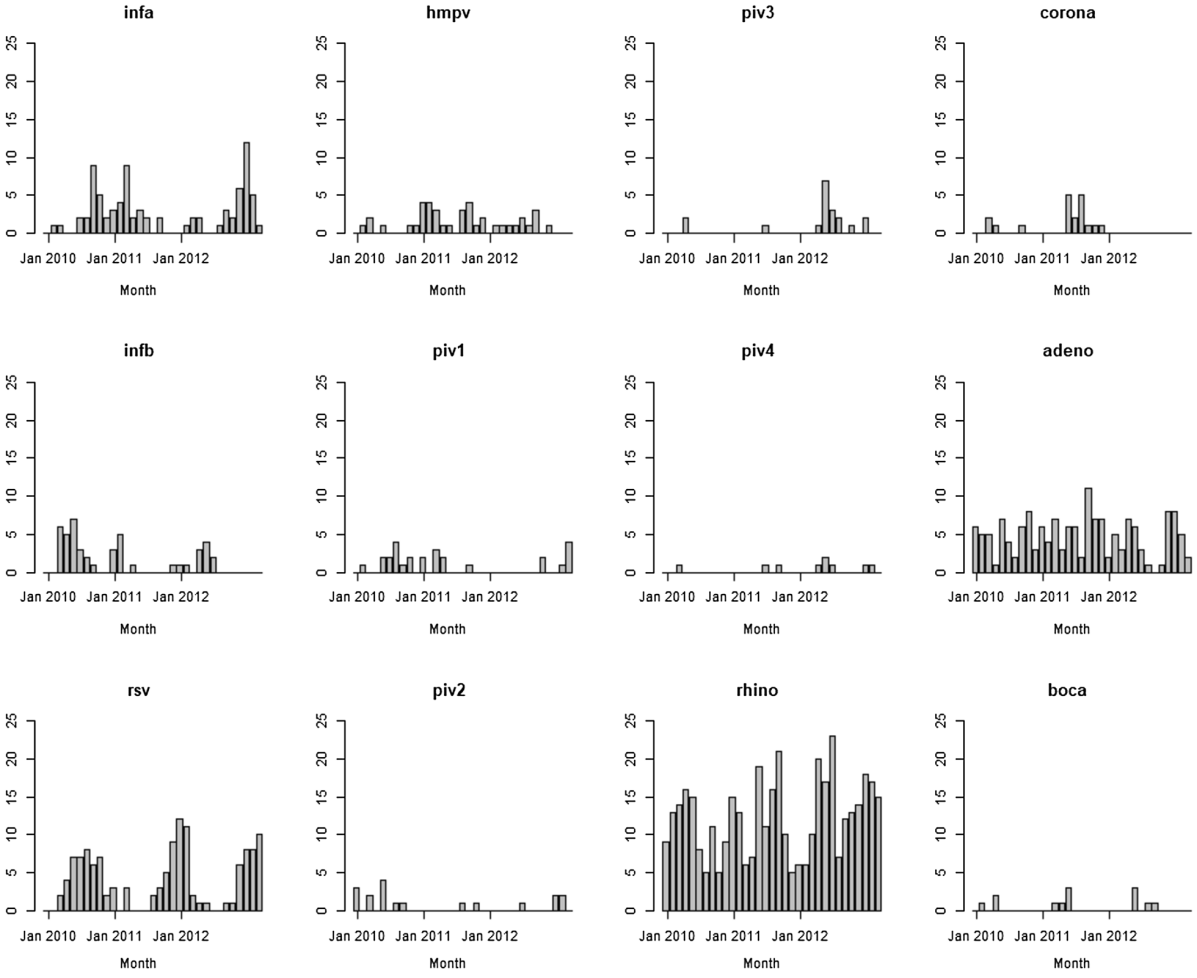
Monthly number of respiratory viruses identified in polyclinics. infa, influenza type **A**. infb, influenza type **B**. rsv, respiratory syncytial virus. hmpv, human metapneumovirus. piv1–4, parainfluenza virus serotypes 1–4. rhino: rhinovirus. corona: coronavirus. adeno, adenovirus. boca, bocavirus. Each bar indicates the monthly number of viruses identified in policlinics from January 2010 to December 2012

During the study period, 15,173 patients, including 12,772 (84.2%) children under 5 years of age, visited polyclinics. Among these, 1,428 samples were collected for further virological analysis (Supplementary Fig. 2). Monthly numbers of positive cases are shown in Fig. 3 and the numbers of detected viruses are shown in Supplementary Table 3. Rhinovirus was the most frequently observed pathogen, accounting for 407 cases (28.5%), followed by adenovirus with 157 cases (11.0%), RSV with 129 cases (9.0%), influenza A virus with 82 cases (5.7%), and influenza B virus with 45 cases (3.2%). Co-infection of respiratory viruses were seen in 172 patients. Among them, 150 patients had co-infection of two respiratory viruses, 20 had three respiratory viruses and two had four respiratory viruses. Among the patients with co-infection of respiratory viruses, rhinovirus was the most frequently detected, with 127 (73.8%) samples, followed by adenovirus with 102 samples (59.3%).

### Pathogen-specific incidence of respiratory viruses and health seeking behaviour

**Fig. 4:**
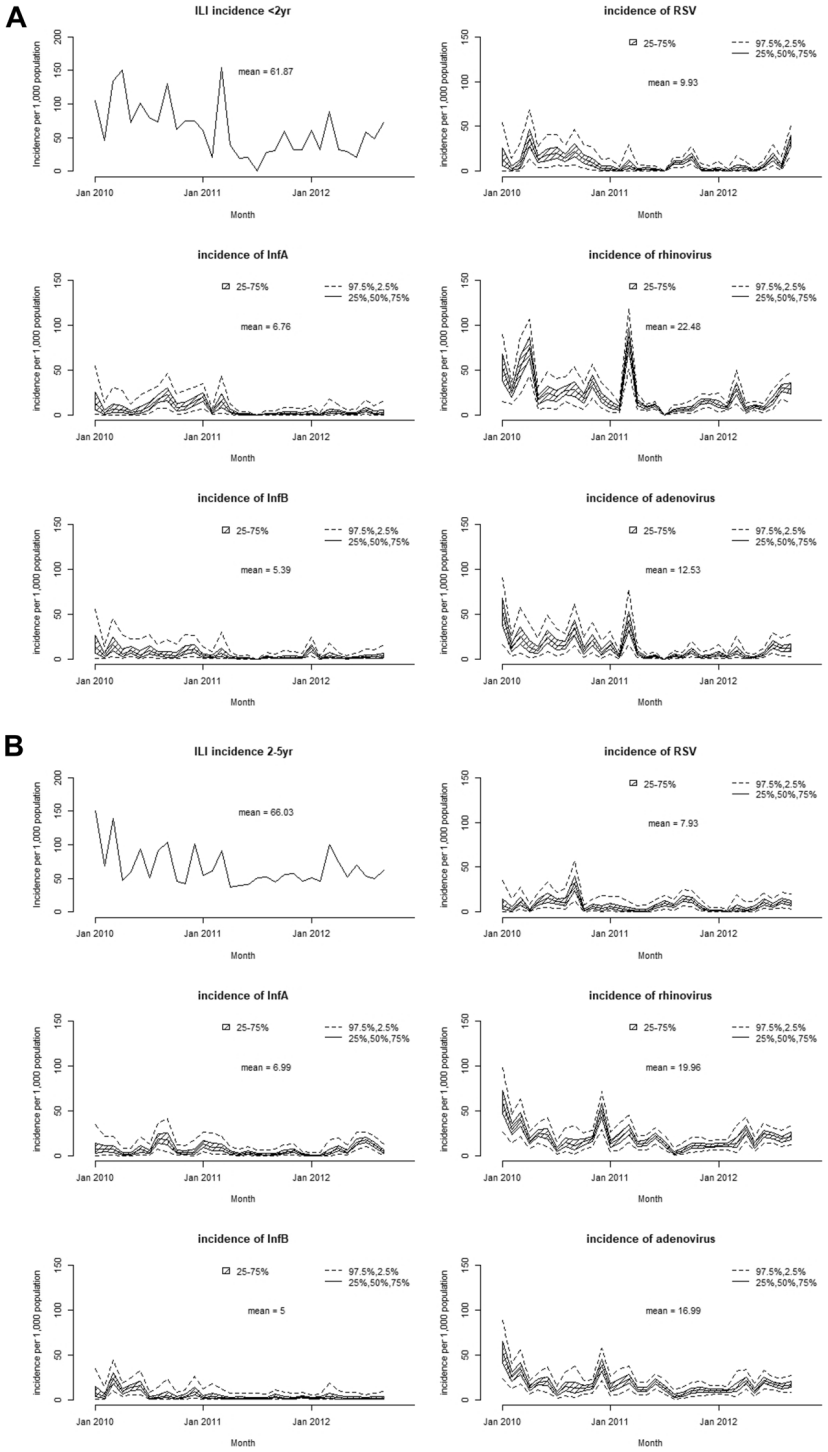
Monthly pathogen-specific incidence per 1000 population of ILI. **A** incidence in < 2 years. **B** incidence in 2–5 years. InfA, influenza type A. infB, influenza type B. RSV, respiratory syncytial virus. Left upper figure showed incidence of ILI in each age group from Jan 2010 to Dec 2012. The other figures showed pathogen-specific monthly incidences of each pathogen calculated by Bayesian methods. After 1,000 times trials to obtain incidence based on equations, median of incidences were shown in middle undashed line with 25–75% of distribution (shaded area) and 2.5–97.5% (dashed line). Means of median incidences in each month were calculated and shown in middle of figures

The monthly pathogen-specific incidence of major respiratory viruses is shown in Fig. 4. Highest incidences were observed in rhinovirus average around 20 episodes/months/1000 population, which raised up to 85.3 (95%CI 50.5–118.4) episodes/months/1000 population in maximum amongst children under 2 years. Adenovirus, RSV, Influenza A virus, and Influenza B virus marked high incidence in the order next to rhinovirus. No significant differences were observed between two age group.

Distinct seasonality was observed in influenza A virus and influenza B virus and RSV. Influenza A virus outbreaks were observed in August–September 2010, December 2010 to January 2011, June 2012, and August 2012. Influenza B virus outbreaks were observed in March–May 2010 and December 2010. Outbreaks of RSV were observed in April–August 2010, July–October 2011, and June–September 2012. In the rainy season, June–September, positive cases of influenza A and RSV increased but those of influenza B decreased.

Total 15,173 patients visited polyclinics −1 and −2 from target population with symptoms of ILI. Among them, the number of visitors under 2 years was 9344(61.5%) and 2–5 years was 3428(22.6%). Randomly selected 1,428 samples were tested for respiratory viruses (Supp. Table 3).

During RSV outbreak months, patients with ILI symptoms in the age group under 2 years tended to visit healthcare facilities [adjusted odds 2.0 (95%CI 1.2–3.5)] compared with those during non-outbreak months, whereas less patients in age groups of 5–14 and 15–29 years visited healthcare facilities. Among the age group of 45–59 years, the proportion of patients with ILI symptoms who visited healthcare facilities increased in influenza B virus outbreak months but decreased in influenza A virus outbreak months, with adjusted odds ratio of 3.0 (95%CI 1.5–5.8) and 0.4 (95%CI 0.2–0.8), respectively (Table 2).

**Table 2:** Adjusted odds ratio of any healthcare facility visit during the outbreak of specific viruses

Outbreak season of each virus	Total	HC visit*	(%)	Crude OR	(95%CI)	*p* value	aOR⁑	(95%CI)	*p* value
0–1 years	289	189	(65.4)						
Influenza A	56	36	(64.3)	0.934	(0.510–1.709)	0.824	0.868	(0.470–1.604)	0.651
Influenza B	58	37	(63.8)	0.909	(0.501–1.649)	0.754	0.771	(0.417–1.428)	0.408
RSV	96	72	(75.0)	1.927	(1.122–3.309)	**0.018**	2.021	(1.162–3.512)	**0.013**
2–4	534	301	(56.4)						
Influenza A	110	53	(48.2)	0.661	(0.435–1.005)	0.053	0.654	(0.425–1.006)	0.053
Influenza B	74	40	(54.1)	0.896	(0.549–1.462)	0.659	0.930	(0.568–1.523)	0.774
RSV	164	92	(56.1)	0.984	(0.680–1.423)	0.930	1.069	(0.731–1.564)	0.730
5–14	913	284	(31.1)						
Influenza A	170	53	(31.2)	1.008	(0.704–1.443)	0.965	1.142	(0.785–1.661)	0.487
Influenza B	106	38	(35.8)	1.280	(0.839–1.953)	0.251	1.286	(0.839–1.971)	0.249
RSV	280	68	(24.3)	0.622	(0.452–0.854)	**0.003**	0.603	(0.434–0.837)	**0.002**
15–29	578	123	(21.3)						
Influenza A	83	11	(13.3)	0.541	(0.280–1.043)	0.066	0.626	(0.320–1.227)	0.173
Influenza B	75	20	(26.7)	1.429	(0.824–2.479)	0.204	1.614	(0.920–2.833)	0.095
RSV	220	35	(15.9)	0.585	(0.380–0.901)	**0.015**	0.597	(0.382–0.934)	**0.024**
30–44	544	100	(18.4)						
Influenza A	130	25	(19.2)	1.087	(0.660–1.791)	0.744	1.132	(0.664–1.929)	0.649
Influenza B	45	9	(20)	1.162	(0.549–2.457)	0.694	1.183	(0.559–2.504)	0.660
RSV	202	36	(17.8)	0.947	(0.604–1.483)	0.811	0.908	(0.562–1.469)	0.695
45–59	417	84	(16.8)						
Influenza A	101	9	(8.9)	0.443	(0.217–0.903)	**0.025**	0.395	(0.191–0.816)	**0.012**
Influenza B	46	15	(32.6)	2.736	(1.415–5.292)	**0.003**	2.957	(1.508–5.797)	**0.002**
RSV	175	31	(17.7)	1.114	(0.687–1.808)	0.661	1.238	(0.753–2.036)	0.399
60 + yr	338	56	(16.6)						
Influenza A	75	11	(14.7)	0.856	(0.423–1.731)	0.666	0.865	(0.420–1.780)	0.693
Influenza B	42	5	(11.9)	0.699	(0.272–1.797)	0.457	0.707	(0.276–1.814)	0.471
RSV	129	21	(16.3)	0.974	(0.542–1.751)	0.930	1.006	(0.552–1.836)	0.983

^*^HC, healthcare facility including hospital, polyclinic, private clinic, and community health centre. Odds ratio (OR) were calculated by penalized maximum likelihood regression. (Stata command “*firthlogit*”). Crude OR of each pathogen indicates odds ratio to visit HC in outbreak seasons compared with those in non-outbreak seasons. Reference numbers were omitted. ⁑aOR, adjusted odds ratio. Since outbreak seasons of each pathogen were duplicated in several months, OR were adjusted by being the outbreak season of influenza A and B and RSV to know the independent likelihood of each pathogen

## Discussion

Estimating the pathogen-specific incidence of ILI in the community is challenging due to the limitations of data sources. Researches based on health care facilities might be underestimated, which do not include cases just taking medicine and staying home [28]. Incidences of some respiratory virus infections commonly diagnosed in clinical settings such as influenza RSV and coronavirus disease 2019 (COVID-19) were well reported but other respiratory viruses [7]. Sylvia T, et al. reported highest incidence in rhinovirus as 29.78 per 100 population/year equal to 24.82 per 1,000 population per month, which is in agreement to this study [18]. Our result of adenovirus as second most identified pathogen was not compatible in other studies, which indicates that endemic viruses in the communities would be varied depends on the area and timing of the studies. [18, 22]

During the COVID-19 pandemic, various diagnostic tools were developed for COVID-19, but not for other respiratory viruses. The combination of community surveillance using calendar-based records and molecular biological surveillance in clinics enabled us to estimate the community burden of each respiratory virus. Children under 5 years tended to visit healthcare facilities rather than just staying home.

Rhinovirus is one of the leading causes of viral bronchiolitis in infants and the most common virus associated with wheezing in children aged 1–2 years. [23] Symptoms of ILI caused by rhinovirus infection are relatively frequent but otherwise a mild, self-limited syndrome. Therefore, its importance as a possible causal factor of severe illness has often been neglected. Multiple infections including rhinovirus were observed in numbers of cases in our surveillance. This study did not analyse the severity of ILI cases. However, previous research has shown that co-infections involving respiratory syncytial virus (RSV) and other pathogens can exacerbate respiratory illness severity by intensifying inflammatory responses [11, 27]. While we did not assess disease severity in our cohort, these findings suggest that co-infections may contribute to more severe outcomes. It is also possible that factors beyond severity, such as the timing of viral outbreaks or accessibility of healthcare facilities, influenced healthcare-seeking behaviour in this study. Further investigation is warranted to explore how these factors interact with severity to shape healthcare utilization patterns.

There were several surges of ILI incidence linked with all age groups, although the highest incidences were in the youngest age group of under 2 years. This might indicate that most ILI symptoms started from the younger generation and spread to the older groups. This study revealed that those surges could not be explained by a single pathogen but a complex of multiple pathogens.

Transmission of respiratory infection is influenced by temperature and humidity [29]. In the northern hemisphere, RSV activity starts in July in tropical areas and later with increasing latitude. In this study, the positive ratio of influenza A and RSV increased in the rainy season, whereas rhinovirus and adenovirus were observed all year, consistent with previous reports. [25, 29, 30]

During RSV outbreak seasons, there were increased numbers of visits to healthcare facilities for the age group younger than 2 years, possibly indicating that severe symptoms with RSV infection leads their carers to take infected children to healthcare facilities rather than just staying home or giving medicine from drug stores.

During influenza B outbreak months, healthcare-seeking behaviour among adults was higher than during influenza A outbreaks. One possible explanation for this observation is the lower incidence of influenza B compared to influenza A in prior seasons, which may result in reduced population immunity against influenza B among adults. This decreased immunity could lead to more pronounced symptoms when exposed to influenza B, prompting higher rates of healthcare-seeking behaviour. Future studies should investigate the immunological profiles and co-infection dynamics in adult populations during influenza outbreaks.

There were several limitations in this study. First, viruses that cause ILI symptoms in the sample population and those observed in polyclinics could differ. We could not get samples from the persons with ILI symptoms in the community in our study setting. Instead, we collected the samples in polyclinics and estimated the community circulating viruses by random sampling from the patients with ILI symptoms. While many common pathogens were observed across age groups in a similar community-based study in Laos [31], differences in pathogen distribution by age highlight the limitation of generalizing pathogen-specific ILI incidence from children under five to the entire population in this study. The Bayesian method enabled us to estimate positive rates for each pathogen with confidence intervals. According to our surveillance, a higher proportion of younger age groups with ILI symptoms visited polyclinics, so we believe that virus detection in polyclinics reflected the circulating viruses in the target communities.

Second, calendar-style health recording may lead to recall bias. This method was effective for daily-based recording; however, when participants forgot to record on time, our research staff needed to ask them to recall ILI events, which may have introduced recall bias. This issue could have been more pronounced in elderly-only households due to potential memory issues. However, such households were rare in this study, with only two identified: one consisting of a couple aged 73 and 78 years, and another with a 79-year-old individual living alone who dropped out in the first 3 months due to the occupant’s death. Given the rarity of such cases, the overall impact on our findings was minimal.

In the beginning of the study, participants were motivated and diligent in recording events, but in the late phase, reporting of ILI symptoms declined, potentially leading to an underestimation of ILI incidence during that period. To address these challenges, healthcare workers conducted monthly or bi-monthly visits to review and update household health calendars, which helped ensure data accuracy and mitigate recall bias.

Second, calendar-style health recording may lead to recall bias. This method was good for daily-based recording; however, when participants forgot to record on time, our research staff needed to ask them to recall ILI events. In the beginning of the study, participants knew the importance of the study and were keen to record the events, but in the late phase, reporting of ILI symptoms declined. Therefore, ILI incidence in the late phase might be underestimated.

Although this study detected 13 respiratory viruses, we focused on rhinovirus, adenovirus, RSV, influenza A, and influenza B for pathogen-specific incidence analysis. The other viruses had a low detection frequency, making it challenging to analyse their seasonality or outbreak patterns reliably. This limitation highlights the need for larger-scale studies or extended monitoring periods to capture the epidemiological dynamics of less frequently detected viruses.

Even with these limitations, this study is valuable in showing the community burden of respiratory viruses in the pre-COVID-19 pandemic era. High incidences of rhinovirus and adenovirus recall us their transmissibility. Continuous monitoring of respiratory viruses in community is required for outbreak preparedness.

## Supplementary Information

Additional file 1.

## Data Availability

No datasets were generated or analysed during the current study.
