# Supplementary material for: Community-based pathogen-specific incidence of influenza-like illness due to respiratory viruses in South-central Vietnam in 2009–2012: after a pandemic of influenza A viruses
Source: Trop Med Health. 2025 Apr 10;53:51. doi: 10.1186/s41182-025-00711-x (PMC11984152; doi:10.1186/s41182-025-00711-x)
Supplement: Supplementary file 1 — Additional file 1. [file 41182_2025_711_MOESM1_ESM.docx]

**Title:**

**Community-based pathogen-specific incidence of influenza-like illness due to respiratory viruses in South-central Vietnam in 2009–2012, after a pandemic of influenza A viruses**

**Supplementary tables and figures**

| **Supplementary table 1. Characteristics of selected household and members.** | | | | |
| --- | --- | --- | --- | --- |
|  | Beginning　of the surveillance | | End of the surveillance | |
|  | (%) | | (%) | |
| **Household numbers** | 1,000 |  | 989 |  |
| Commune |  |  |  |  |
| Vinh Phuoc | 247 | (25) | 242 | (24) |
| Vinh Hiep | 250 | (25) | 250 | (25) |
| Vinh Thanh | 250 | (25) | 247 | (25) |
| Vinh Hoa | 253 | (25) | 250 | (25) |
| Household size |  |  |  |  |
| ≤4 persons | 435 | (44) | 534 | (54) |
| 5–6 persons | 290 | (29) | 293 | (30) |
| ≥7 persons | 275 | (28) | 162 | (16) |
| **Household members** | 4,716 |  | 4,755 |  |
| Commune |  |  |  |  |
| Vinh Phuoc | 1,307 | (28) | 1,291 | (27) |
| Vinh Hiep | 1,087 | (23) | 1,089 | (23) |
| Vinh Thanh | 1,234 | (26) | 1,271 | (27) |
| Vinh Hoa | 1,088 | (23) | 1,104 | (23) |
| Sex |  |  |  |  |
| Female | 2405 | (51) | 2425 | (51) |
| Age (years) |  |  |  |  |
| Mean ± SD | 32.0 ± 20.0 |  | 33.7 ± 20.3 |  |
| Median (IQR) | 30 (16 – 46) |  | 32 (18 – 48) |  |
| Age group (years) |  |  |  |  |
| <2 | 147 | (3) | 97 | (2) |
| 2–4 | 199 | (4) | 194 | (4) |
| 5–14 | 690 | (15) | 654 | (14) |
| 15–29 | 1,249 | (26) | 1,223 | (26) |
| 30–44 | 1,158 | (25) | 1,183 | (25) |
| 45–59 | 824 | (17) | 888 | (19) |
| 60+ | 449 | (10) | 516 | (11) |
| Four communes, Vinh Phuoc, Vinh Hiep, Vinh Thanh and Vinh Hoa were selected for the study. On the beginning of the surveillance in September 2009, number of households were 1,000 including 4,716 household members. At the end of the surveillance in December 2012, number of households were decreased but household members were slightly increased. Mean age was increased by 1.7 years. | | | | |

| **Supplementary table 2. Movement of household members during study period** | |
| --- | --- |
| **Movement** | **Number** |
| **New-born** | **148** |
| **Died** | **61** |
| **Moved in** | **116** |
| Khanh Hoa | 17 |
| Nha Trang | 54 |
| Other province | 33 |
| Overseas | 2 |
| Unknown | 10 |
| **Moved out** | **93** |
| Khanh Hoa | 11 |
| Nha Trang | 30 |
| Other provinces | 30 |
| Overseas | 10 |
| Unknown | 12 |
| **Dropped out** | **59** |
| Total increase | 264 |
| Total decrease | 213 |
| **Total change** | **+51** |

| 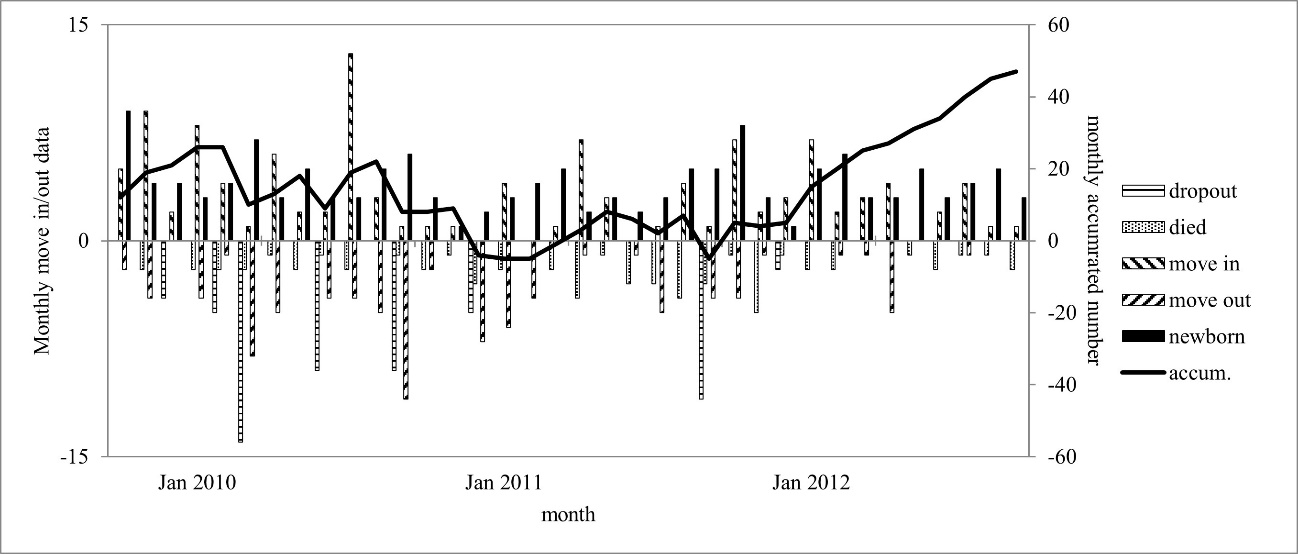 |
| --- |
| **Supplementary figure 1.** Monthly changes of sample population were recorded regarding to self-reports of household members. Persons who moved in households were defined as those who stay more than three weeks. Persons who moved out from households were defined as those who were absent for more than three weeks. |

| 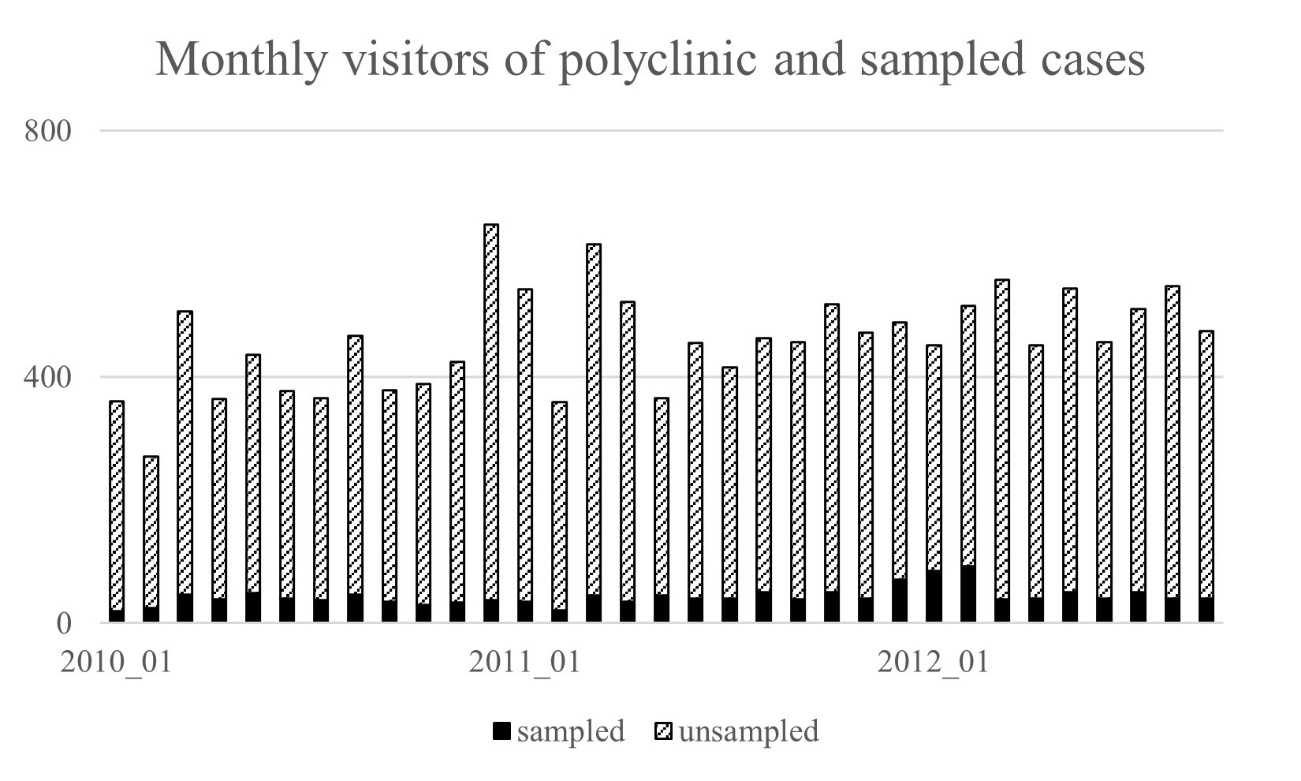 |
| --- |
| **Supplementary figure 2.** |

| **Supplementary table 3. Number of visitors to polyclinics 1 and 2 from target commune and identified viruses** | | |
| --- | --- | --- |
|  | N | (%) |
| Total visitors from target population | 15,173 |  |
| <2 years from target population | 9,344 | (61.5) |
| 2–5 years from target population | 3,428 | (22.6) |
| Total sampled | 1,428 |  |
| Influenza A | 82 | (5.7) |
| Influenza B | 45 | (3.2) |
| RSV | 129 | (9.0) |
| hMPV | 40 | (2.8) |
| PIV1 | 27 | (1.9) |
| PIV2 | 18 | (1.3) |
| PIV3 | 19 | (1.3) |
| PIV4 | 9 | (0.6) |
| Rhinovirus | 407 | (28.5) |
| Coronavirus | 19 | (1.3) |
| Adenovirus | 157 | (11.0) |
| Bocavirus | 13 | (0.9) |
| At least one virus | 769 | (53.8) |
| No virus detected | 659 | (46.2) |
| Multiple infection |  |  |
| 2 viruses | 150 | (10.5) |
| 3 viruses | 20 | (1.4) |
| 4 viruses | 2 | (0.1) |
| RSV, respiratory syncytial virus. PIV 1-4, parainfluenza virus type 1-4. Numbers of children under 5 years old visited polyclinics were showed here. See also supplementary fig.1 for monthly numbers of visitors and those sampled. | | |
